# Virtual Reality App for Treating Eating Behavior in Eating Disorders: Development and Usability Study

**DOI:** 10.2196/24998

**Published:** 2021-04-13

**Authors:** Billy Sundström Langlet, Dorothy Odegi, Modjtaba Zandian, Jenny Nolstam, Per Södersten, Cecilia Bergh

**Affiliations:** 1 Division of Clinical Geriatrics, Center for Alzheimer Research Department of Neurobiology, Care Sciences and Society Karolinska Institutet Stockholm Sweden; 2 Mandometer Clinic Stockholm Sweden

**Keywords:** feeding and eating disorders, anorexia nervosa, bulimia nervosa, binge eating disorder, immersive virtual reality, eating disorders, virtual reality

## Abstract

**Background:**

Anorexia nervosa is one of the more severe eating disorders, which is characterized by reduced food intake, leading to emaciation and psychological maladjustment. Treatment outcomes are often discouraging, with most interventions displaying a recovery rate below 50%, a dropout rate from 20% to 50%, and a high risk of relapse. Patients with anorexia nervosa often display anxiety and aversive behaviors toward food. Virtual reality has been successful in treating vertigo, anxiety disorder, and posttraumatic stress syndrome, and could potentially be used as an aid in treating eating disorders.

**Objective:**

The aim of this study was to evaluate the feasibility and usability of an immersive virtual reality technology administered through an app for use by patients with eating disorders.

**Methods:**

Twenty-six participants, including 19 eating disorder clinic personnel and 5 information technology personnel, were recruited through emails and personal invitations. Participants handled virtual food and utensils on an app using immersive virtual reality technology comprising a headset and two hand controllers. In the app, the participants learned about the available actions through a tutorial and they were introduced to a food challenge. The challenge consisted of a meal type (meatballs, potatoes, sauce, and lingonberries) that is typically difficult for patients with anorexia nervosa to eat in real life. Participants were instructed, via visual feedback from the app, to eat at a healthy rate, which is also a challenge for patients. Participants rated the feasibility and usability of the app by responding to the mHealth Evidence Reporting and Assessment checklist, the 10-item System Usability Scale, and the 20-point heuristic evaluation questionnaire. A cognitive walkthrough was performed using video recordings of participant interactions in the virtual environment.

**Results:**

The mean age of participants was 37.9 (SD 9.7) years. Half of the participants had previous experience with virtual reality. Answers to the mHealth Evidence Reporting and Assessment checklist suggested that implementation of the app would face minor infrastructural, technological, interoperability, financial, and adoption problems. There was some disagreement on intervention delivery, specifically regarding frequency of use; however, most of the participants agreed that the app should be used at least once per week. The app received a mean score of 73.4 (range 55-90), earning an overall “good” rating. The mean score of single items of the heuristic evaluation questionnaire was 3.6 out of 5. The lowest score (2.6) was given to the “accuracy” item. During the cognitive walkthrough, 32% of the participants displayed difficulty in understanding what to do at the initial selection screen. However, after passing the selection screen, all participants understood how to progress through the tasks.

**Conclusions:**

Participants found the app to be usable and eating disorder personnel were positive regarding its fit with current treatment methods. Along with the food item challenges in the current app, participants considered that the app requires improvement to offer environmental and social (eg, crowded room vs eating alone) challenges.

## Introduction

Anorexia nervosa is an eating disorder characterized by restriction of energy intake, leading to a significantly low body weight, intense fear of gaining weight, disturbed body perception, and lack of insight into the seriousness of the disorder [1]. At least 90% of individuals with anorexia nervosa are women, with 40% of the identified patients ranging between 15 and 19 years of age [2,3]. According to the Diagnostic and Statistical Manual of Mental Disorders IV, the lifetime prevalence of anorexia nervosa varies from 0.3% to 1.0%, and the mean crude mortality rate is 5% per decade [4,5], varying from 0% to 15.6% [6,7]. Anorexia nervosa is associated with physical problems such as anemia, reduced brain volume, infertility, altered hormonal balance, loss of muscle mass, and osteoporosis. However, most of these physical and mental problems normalize with weight regain [8].

Long-term outcomes in anorexia nervosa treatments are often discouraging. A 2002 review concluded that only 46.9% of anorexia nervosa patients reached full recovery, 33.5% improved, and the disorder became chronic in 20.8% of cases [9]. In 2020, similar outcomes were found in anorexia nervosa patients receiving treatments consisting of eating disorder–focused structured individual therapies [10]. In addition, studies frequently report poor long-term treatment outcomes with high relapse rates [11], dropout rates ranging from 20.2% to 49.6% [12], and a propensity for the anorexia nervosa disorder to transition into other eating disorders [13]. Better treatment outcomes could be achieved when one part of the treatment consists of normalizing eating behavior through eat training [14]. Treatment based on this method typically begins after establishing a baseline eating behavior for each patient by having them eat food on a medical device (Mandometer) that records the cumulative food intake (in grams). In this treatment, patients eat their meals on the Mandometer following a reference curve for food intake over time that is displayed on their own smartphone. The food intake quantity is initially based on the baseline measure but is updated depending on the rate of recovery of the patient [15]. Another part of eat training consists of reducing food avoidance and apprehension around food, which are the key factors responsible for maintaining the starved state [16]. Virtual reality (VR) technology could offer an alternative approach for handling food and to practice eat training in a treatment setting [17].

VR treatment involves immersing an individual in a computer-generated 3D world that is customized according to treatment needs, where the individual can be safely exposed to stressors. Additional benefits of using VR are that it enables repetition as well as exposure control (internal validity) and has high generalizability to other contexts (external validity). Recent technological advances have greatly reduced the cost of VR technology, thereby increasing its scalability [18]. To date, intervention studies that employed VR have been successful in treating posttraumatic stress syndrome, anxiety syndrome, and smoking [19,20]. In the eating disorder context, most studies that have employed VR used the technology as an assessment tool for exploring body image perception, and the response of virtual foods and environments [21]. One study demonstrated the ecological validity of the approach, with VR food eliciting similar responses as real food in patients with eating disorders [22]. Most of the eating disorder interventions employing VR have aimed to correct a distorted body image [23]. However, VR exposure therapy has also been reported to reduce the anxiety response to food in patients with bulimia nervosa [24]. VR therapy may also be more acceptable to eating disorder patients than other treatment forms. In a study on phobias, VR exposure was more likely to be selected (76% of participants chose VR) and had lower refusal rates than in vivo exposure (3% vs 27%, respectively) [25]. These findings suggest that exposure therapy via VR has internal, external, and ecological validity in the target group, indicating that it can be effective in treating bulimia nervosa and perhaps also anorexia nervosa, and may be more acceptable for end users (patients with eating disorders) [25].

Evaluation of interventions is common; however, the quality of these studies is often poor, which leads to incorrect implementations and findings that are not reproducible [26]. The first step of establishing digital health interventions is usually to gather the functional requirements for development and testing [27]. The mHealth Evidence Reporting and Assessment (mERA) checklist is a useful method for reporting on digital interventions, which has the benefit of providing recommendations for reporting the feasibility of intervention strategies [27]. Another key factor for the successful adoption of a new technology is usability [28], which was defined as “the extent to which a product can be used to achieve specified goals with effectiveness, efficiency and satisfaction in a specified context of use” [29]. To ensure the acceptance and attitude of patients and clinicians, as well as to enhance well-being, reduce risk of harm, and increase accessibility for patients, health technologies should be appropriately designed to the end users’ needs before they are deployed as health interventions [30,31]. One review suggests using multiple methods in performing usability evaluations [29]. Another study found that the System Usability Scale (SUS) was the most common questionnaire used in such evaluations, and recommended the use of quantitative evaluation methods but concluded that further research is needed to identify which methods are best suited for different patient groups [28].

The aim of this study was to investigate the perceived usability of a newly developed VR app that simulates eat training and is intended for use in an eating disorder intervention study. As a first step in this development, usability was evaluated by staff involved in eating disorder treatment.

## Methods

### Participants

To be eligible for participation in the study, the individual had to be working in an eating disorder clinic and have daily interactions with eating disorder patients as a clinician or physician (eating disorder personnel) or as part of information technology (IT) service (IT personnel). Individuals were invited to participate in the study through emails and personal invitations. Eating disorder patients were not approached at this stage because of the potential risk that participation would result in poorer treatment outcomes and the assessment that many of the questions related to intervention delivery would be difficult for patients to answer.

### Technology

The HTC VIVE VR system (HTC) was used as the immersive VR technology in this study, consisting of a headset (connected to a computer) through which the VR environment could be viewed, two hand controllers that enabled interaction with the VR environment, and two base stations that enabled motion tracking. To ensure proper performance, the room size should be at least 1.5×2.0 meters. The computer used a NVIDIA GeForce GTX 1060 graphic card, an Intel Core i5-4590 processor, 8 GB of RAM, and the Windows 7 operating system. The software was developed in the Unity engine and ran on the digital game distribution platform Steam (Valve Corporation), using the SteamVR app.

### Instruments

Four methods were used for the usability evaluation: the mERA checklist, 10-Item SUS, 20-item heuristic evaluation questionnaire, and a cognitive walkthrough. Rather than being forced to respond to each question, participants could leave questions blank if unable to respond.

The mERA checklist is a 16-item checklist that aims to standardize reporting, and enables quick assessment of eHealth and mobile health apps. Adoption of this checklist is meant to highlight issues of generalizability and rigor in reporting and improving transparency [27]. The procedure and app allowed the researchers to objectively evaluate the infrastructure and technological platform, and to make a cost assessment (items 1, 2, and 9). A one-on-one interview was held with each participant regarding the items that could not be objectively evaluated (items 3, 4, 5, 7, 8, 10, 11, and 12). The specific question for each item was whether the participant could foresee any obstacles related to the item (eg, how easy it would be to integrate the app and VR system with already existing health care systems) if the app and VR system were to be used in an identical fashion to how eat training is currently performed in a clinical setting. Questions on interoperability and adaptability were answered by all participants (items 3 and 12), whereas questions on intervention delivery, content testing, accessibility, adoption, scalability, and user feedback were answered only by the eating disorder personnel (items 4, 5, 7, 8, 10, and 11). Since the app was at a formative stage, five questions on the checklist were not applicable (items 6, 13, 14, 15, and 16).

The SUS is a validated tool that is widely used to assess the perceived usability of a system. It consists of 10 statements such as “I found the system unnecessarily complex” and “I felt very confident using the system,” which are answered on a 5-point Likert scale with verbal anchors at the extremes from 0 (“strongly disagree”) to 4 (“strongly agree”). The sum result of the SUS is a score ranging from 0 to 100 [32].

The cognitive walkthrough is a usability evaluation method in which evaluators work through a series of tasks required by the system asking a set of questions from the perspective of the user [33]. The purpose of the cognitive walkthrough is to evaluate the ease of use of an interface design to new or infrequent users. In this study, the following four steps of human-computer interaction were evaluated: (1) goals to be completed in the system, (2) determination of currently available actions, (3) selection of actions to be taken, and (4) performance of the tasks and evaluation of the feedback given by the system. We determined whether a participant managed to perform a step for each task by reviewing video recordings of their interaction with the app (eg, Task 3. Read instructions, grab spoon, pick up meatballs, place meatballs on plate, and move to next task; see Multimedia Appendix 1). Video recordings of actions performed by the user in the app were made using the inherent Steam streaming software. The video recordings were complemented with audio recordings on a smartphone.

The heuristic evaluation is a method for evaluating the usability of computer software, focusing on identifying problems with the user interface. As the name implies, this questionnaire evaluates recognized usability principles (heuristics), constituting one of the more informal methods of human-computer interaction. The heuristic evaluation questionnaire used in this study was based on the Weinschenk and Barker [34] classification, which consist of 20 items, for example “User support: does the application provide additional assistance as needed or requested?” Each item was answered on a 5-point Likert scale with verbal anchors at the extremes from 1 (“strongly disagree”) to 5 (“strongly agree”).

### Procedure

**Figure 1:**
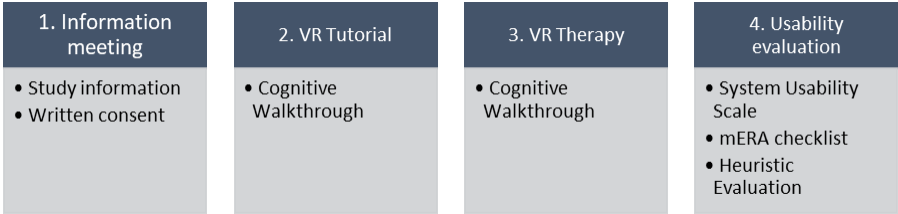
Study protocol presented in chronological order from left to right. VR: virtual reality; mERA: mHealth Evidence Reporting and Assessment.

Each participant attended an information meeting, including a VR tutorial, VR eat training, and a usability evaluation (Figure 1). They were informed of the study and encouraged to ask questions, after which they signed a written consent form if they agreed to participate.

**Figure 2:**
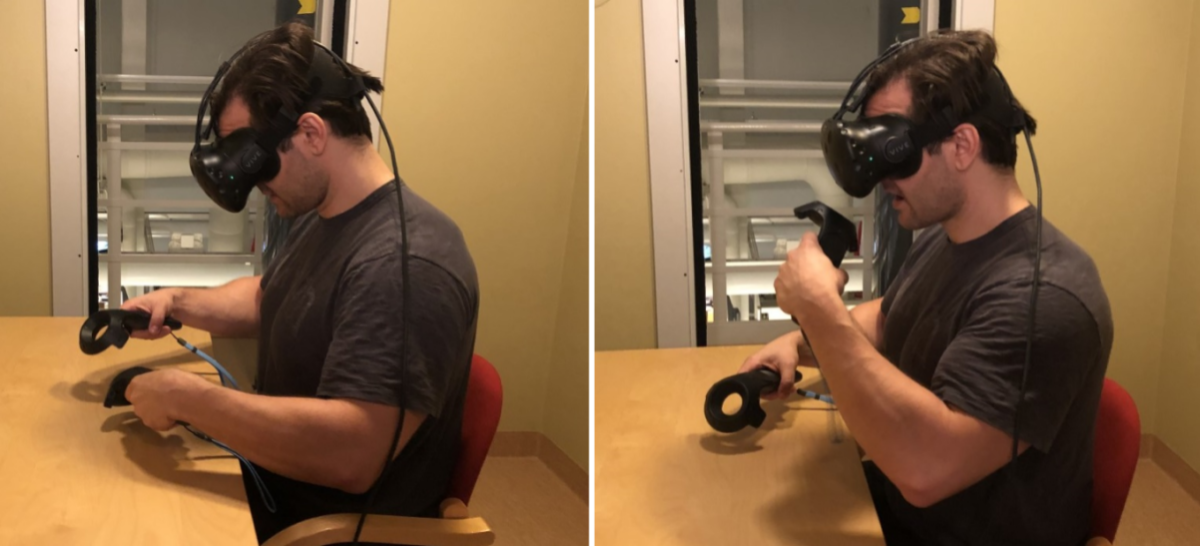
Participant interacting with virtual food on the plate (left) and participant eating virtual food (right).

The participants were then taken to the VR lab to familiarize themselves with the VR equipment and environment (Figure 2). The VR lab tutorial started with calibration of the app so that a virtual table and chair were in the same position as a real table and chair in the VR lab. The participant was seated on the real chair in front of the real table and was then fitted with the VR equipment. At this point, video and voice recording features were initiated. In the VR environment, the controllers were represented by hands. The tutorial was built in steps, with each step being supported by instructions on what tasks to perform to meet the goal to proceed to the next step, which were displayed on a virtual smart tablet present in the VR environment.

**Figure 3:**
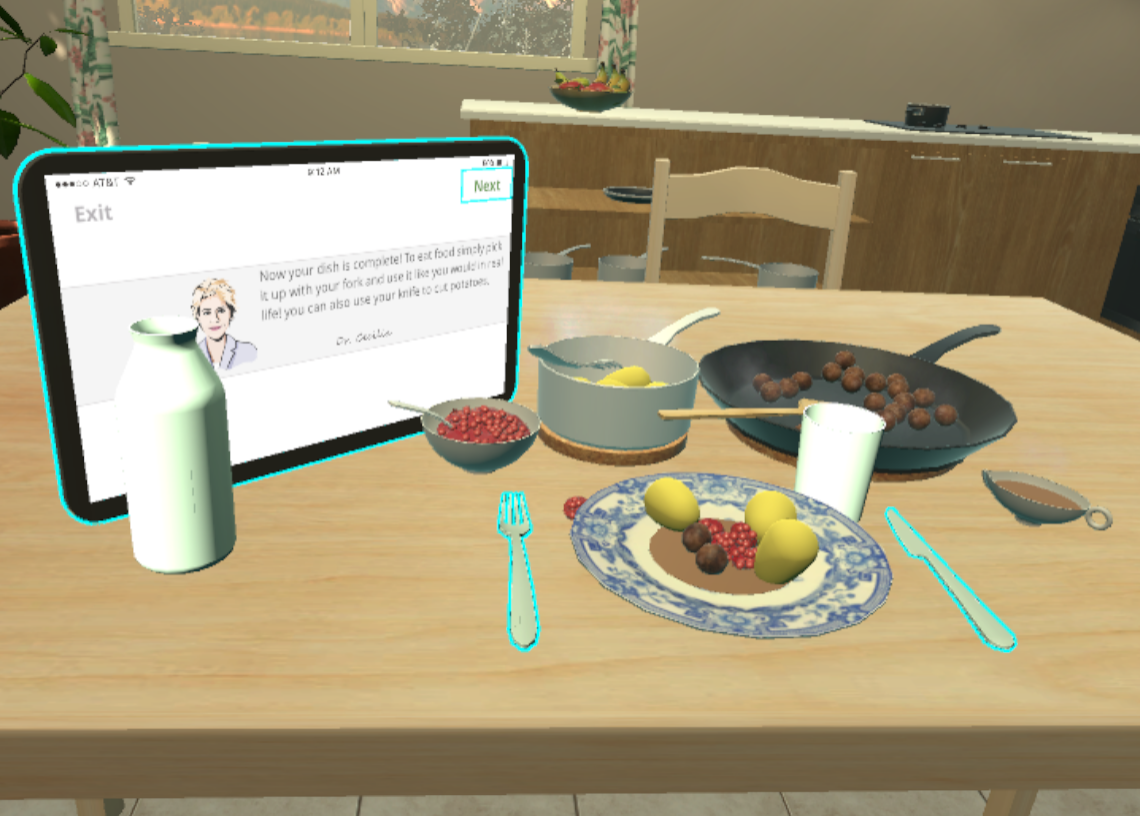
Free interaction with food during the tutorial step of the app.

The success of each task was evaluated based on the performance of the participants (Figure 3). In the first step, participants used the controller to interact with the “next” button on the virtual smart tablet. Each subsequent step ended by pressing the “next” button. In the second step, a plate appeared on the table and the participant placed the plate at a specific position on the table. In the third step, a pan of meatballs and a spoon appeared; participants had to then transfer the meatballs from the pan to the plate using the spoon. In the fourth step, a pot of potatoes and a fork appeared; participants had to transfer the potatoes from the pot to the plate using the fork. In the fifth step, a sauce boat filled with gravy appeared; participants had to pour gravy from the sauce boat over the plate. In the sixth step, a bowl of lingonberry jam and a spoon appeared; participants had to transfer the lingonberry jam from the bowl to the plate using the spoon. In the seventh step, a jug of water and a glass appeared; participants had to pour water from the jug into the glass. In the final step of the tutorial, participants interacted freely with the served food, cutting and eating it.

Once familiarized with the app, the participant experienced VR eat training in the same manner as eat training with real food is practiced in the clinic. First, the participant was asked to place a healthy portion of food on the plate. Feedback on how close that portion was to a healthy portion was presented on the computer screen (expressed as percentages). Once the screen showed 100%, the participant started to eat. At this point, the training curve for food intake was displayed on the screen, and as the virtual meal progressed, the virtual food intake also emerged on the screen. Similar to treatment of real eating behavior, the participant tried to eat virtually following the training curve [15]. Once all of the virtual food had been consumed, the duration of the meal, amount of food eaten, and rate of eating were presented on the screen, and the participant was given the option to close the app.

After having experienced both the tutorial and VR eat training session, participants sat down with one researcher to answer the SUS, heuristic evaluation, and mERA checklist questionnaires. Initially, the participants were expected to answer the questions alone. However, it became immediately obvious that some questions required clarification and affirmation by the researcher to confirm proper interpretation. Therefore, a researcher was present in the room when the participant filled in the questionnaires to provide help when needed. After completing the questionnaires, participants received a cinema ticket as a reward and they were thanked for their participation.

### Data Interpretation

Results are expressed as percentage among responding participants. To provide context for the SUS score, the adjective rating scale proposed by Bangor et al [35] was used, where a score of 39.17 to 52.00 is considered “poor,” 52.01 to 72.74 is considered “OK,” 72.75 to 85.57 is considered “good,” and 85.58 to 100.00 is considered “excellent.”

### Ethical Approval

All procedures were approved by the Swedish Ethical Review Authority (dnr 2019-04249) and followed the Helsinki Declaration. Each participant attended an information meeting and provided written consent for participation.

## Results

### Participants

A total of 24 participants (67% women) were recruited, including 19 eating disorder personnel and 5 IT personnel. Their mean age was 37.9 (SD 9.7) years. Eating disorder personnel had worked at an eating disorder clinic for 4.1 (SD 5.7) years. Twelve of the 24 participants (50%) had previously experienced VR, including 42% (8/19) of the clinicians and 80% (4/5) of the IT participants.

IT personnel were only able to answer 20% of the mERA questions intended for them, and these responses were therefore excluded from the analysis of mERA results. The reason given by IT personnel for not being able to answer the questions was that they are not directly involved in the clinical treatment aspects. Eating disorder personnel were able to answer 80% of the mERA questions intended for them and these responses were therefore included in the mERA questionnaire analysis.

### mERA Checklist

#### Item 1: Infrastructure

In Swedish urban environments, the availability of power supplies and network connections is adequate to support the app; however, it is uncertain whether the infrastructure in rural environments would be sufficient. The size of the room required for the equipment makes it possible to implement in any facility. However, when the space is smaller than 1.5×2.0 meters, there is a risk of signals from the controller and headset not being transferred to the base stations, causing the app to pause.

#### Item 2: Technology Platform

The app performed well on a computer with low hardware specifications (see the Technology subsection of the Methods), but hardware requirements may be higher if other apps are expected to run simultaneously.

#### Item 3: Interoperability

All of the responding eating disorder personnel (100%, 10/10) thought that it would be easy to integrate the app with the clinic’s medical record system, but would be more difficult to integrate with the national care information system. The main problem mentioned in relation to the national system is that visual presentations are difficult to implement, but simplified presentation of data would be possible.

#### Item 4: Intervention Delivery

All participants thought that during periods of app use, the frequency should be at least once a week. Participants thought that the current app should be used during treatment, with a higher frequency at the start of treatment. They considered that the addition of difficult environmental and social situations (challenges) would make it usable at a similar frequency throughout treatment.

#### Item 5: Content Testing

All eating disorder personnel thought that the app should complement real eat training using VR to expose patients to food, environment, and social challenges. The addition of challenges would require alterations to the app.

#### Item 7: User Feedback

All responding eating disorder personnel were pleased with the state of the app (100%, 14/14) and thought that future interventions would be easy due to the similarities of the app with current clinical treatment protocols.

#### Item 8: Access of Individual Participants

Nine out of 19 (47%) eating disorder personnel thought that the app was usable for the treatment of all eating disorders; the remainder were unsure but stated no specific barriers to use. Other potential barriers identified were age, culture (availability of only one meal), language (only an English version is currently available), and epilepsy. Overall, 16% (3/19) of the eating disorder personnel expressed a concern that the patients may replace real meals with virtual meals, thereby complicating treatment.

#### Item 9: Cost Assessment

The currently used setup (computer and VR set) costs approximately US $1600, which is affordable for both research and clinical purposes. Based on an intervention protocol of 1 hour per week for each patient, a clinic should be able to run an intervention using two VR devices, which would cost approximately US $3200. There is also the added cost of renting space and a part time clinician.

#### Item 10: Adoption Inputs

Eating disorder personnel thought that the app should be introduced early in treatment and be described as an aid to patients, similar to the medical device (Mandometer) currently used by the clinic, which measures food weight and provides feedback on eating behavior.

#### Item 11: Limitations for Delivery at Scale

Regarding limitations at scale, 32% (6/19) of the participants thought that providing technical support for installing, updating, and using the system could cause problems. Moreover, 21% (4/19) of the participants thought that implementation for clinics using other treatment forms could be problematic. Few mentioned costs (11%, 2/19) and time requirements (5%, 1/19) as potential problems for scaling up.

#### Item 12: Contextual Adaptation

Eating disorder personnel agreed that the most important aspect of the app should be eat training and exposure to food, but commented that including aspects of the environment (eg, school dining hall and birthday party) and social challenges (eg, eating with other people, eating when people are talking) could further improve the app. Other suggestions included reducing anxiety by providing relaxing environments. There was also a suggestion to add a reward system, where patients could score points if they managed to succeed in various challenges.

### SUS Questionnaire

The SUS scores were similar between clinicians and IT participants, with a mean score of 73.4 (SD 9.2) and 73.5 (SD 11.3), respectively (for a total mean score of 73.4, range 55-90), earning an overall “good” rating. Although the sample sizes did not allow for a statistical comparison, the largest discrepancy between single items was found for the question “I think that I would need assistance to be able to use this system,” for which clinicians were less confident that patients would be able to use the app without support (1.3, mean difference –0.9). The highest score was given to the item “I would imagine that most people would learn to use this system very quickly,” with a mean score of 3.5 (maximum score 4).

### Heuristic Evaluation

The mean score of single items of the heuristic evaluation questionnaire was 3.6 out of 5, and was very similar between eating disorder and IT personnel (3.6 and 3.7, respectively). Mean values of specific items ranged from 2.6 to 4.4. Both eating disorder and IT personnel provided low scores on “accuracy,” with a mean score of 2.6 (SD 1.1). Eating disorder personnel also provided a low score on “user support”, with a mean of 2.9 (SD 1.3), whereas the IT personnel provided low scores on “flexibility,” with a mean of 2.0 (SD 0.7).

### Cognitive Walkthrough

The only task in which participants faced problems in understanding the goals to be completed in the app was at the initial selection screen (step 1), where 29% (7/24) of the participants needed instructions on how to select between the tutorial and eat training part of the app. All participants were able to determine the available actions (step 2) and select which actions to take (step 3). All participants required further instructions at least once when trying to perform the tasks (step 4). Based on the instructions required for the participant to complete the task, six problems were identified: (1) handling utensils that used “magnetic” properties (ie, where the food latched onto the utensil based on proximity) rather than gravity to interact with food (serving potatoes and lingonberries) was the most challenging issue; (2) the utensils had specific attributes tied to them (ie, the fork was used for picking up food and the knife was used for cutting), which created problems for participants who tried to pick things up with their hands in some cases (controllers not holding utensils) or used both utensils for picking up food; (3) it was not possible to divide the meatballs and the potatoes more than twice, which confused some participants; (4) only one food item (eg, potatoes or meatballs) could be placed on the fork at a time, and most participants tried to put both on the fork together initially; (5) when drinking, there was a risk of hitting the VR headset with the controller because the water glass was held closer to the controller compared with the fork holding food; and (6) when the VR environment (SteamVR) was improperly calibrated, it became difficult to interact with the items.

## Discussion

Using the app tested in this study in a clinical setting would likely only face minor infrastructural, technological, interoperability, financial, or adoption problems. Eating disorder personnel seemed to be positive at the prospect of using the app in the clinic. However, there was some disagreement on the protocol of intervention delivery. SUS scores suggested that the system is passable with room for improvement. The heuristics of the user interface was acceptable, identifying user support, accuracy, and flexibility as potential weaknesses. Video and audio recordings of users’ interaction with the app suggest that users knew the goal of each step of the app and understood when they had successfully completed each step. A suggestion for improvement of the app was to add environmental and social stressors, and to use them in a similar manner as the food stressors in the current app.

Despite its early stage of development, compared with other systems, the app performed above average on the SUS [35]. These findings are in line with answers on the mERA checklist, heuristic evaluation, and observations from the cognitive walkthrough. The results also indicate a willingness by clinicians to use the app in treatment, which is important to ensure proper intervention fidelity [36]. Regarding the heuristic evaluation, the lowest scores were given to “accuracy,” “flexibility,” and “user support.” The low “accuracy” scores seem to have stemmed from difficulty in using the fork. This may have been caused by different types of cutlery having different functions associated with them. In practice, this meant that interactions with the fork worked on proximity, similar to a magnet, whereas interaction with the spoon was based on the regular gravitation properties of objects. Both methods of interaction are often used in VR apps, but mixing the two likely resulted in reduced accuracy. The low flexibility score indicates a need for user customization. The next version of the app should therefore include visual and audio information in both Swedish and English, as well as a more varied selection of cutlery (eg, spoon and fork) and dishes. The low score on user support suggests the requirement of technical support during the intervention. One problem addressed by eating disorder personnel is that there does not seem to be a safe way for individuals with photosensitive epilepsy to use VR equipment. However, this group only accounts for approximately 10% of epilepsy cases in the age range of 7-19 years [37]. Another worry of eating disorder personnel was that patients would replace real meals with virtual meals. Addressing this concern is beyond the scope of this study but should be considered when conducting VR interventions for eating disorder patients.

Responses to the mERA checklist suggested that infrastructural requirements were low using the current VR technology (HTC VIVE). However, some clinics could face issues, especially if rooms serve multiple purposes, which would require the system to be mobile. One solution would be to use more mobile alternatives such as Oculus Quest, which do not require base stations or a computer connection. The low hardware requirements also suggest that most clinics should be able to use the app with their current computers. Even if new computers are purchased for the intervention, the estimated cost of a VR study using current VR technology is low. Given the similarity of the data provided by the app to data already handled by the medical records system of the intended clinics, only minor modifications are required to incorporate the app in treatment. The reason that IT personnel rated the requirement for assistance lower than the eating disorder personnel may be because they were more likely to have used VR before. In the intended intervention, assistance requirements will not be a problem since patients will be assisted by eating disorder personnel. However, if future interventions intend for the app to be used unassisted or outside of the clinic, additional assistance provided by the app is likely required. Despite the concerns that users may need assistance when using the app, the highest usability score was given to the ease of learning how to use the system.

According to participant feedback, the app should be introduced at the beginning of treatment (intervention) and be used in parallel with real-life eat training. The app should be used throughout the treatment course, with a focus on food challenges at the beginning, and environment and social challenges introduced at the end. VR sessions should be administered at least once a week, for at least the duration of a single meal (around 12 minutes), but preferably for a few meal scenarios. In addition, each clinic should have one person on call for technical support when the VR sessions are performed. The proposed intervention protocol has a slightly lower frequency (once per week vs twice) but a longer duration (3 weeks vs treatment duration) than a study on binge eating performed by one of the more prominent groups focusing on VR-based treatment [38]. However, this protocol is similar to the current treatment [15], which eating disorder personnel thought should make implementation easy.

A strength of this study was the high number of eating disorder personnel included, which increases confidence in generalizability of the findings. However, it should be noted that the personnel all had experience in using a similar treatment protocol; thus, clinicians administering other treatment protocols may respond differently. A potential weakness was the use of eating disorder personnel rather than patients with eating disorders. However, many of the questions related to intervention delivery at this early stage of development would be difficult for patients to answer. This, along with the worry that patients may be affected negatively by the app, was why we recruited only eating disorder and IT personnel for this study. After ensuring that the VR technology and the app are safe for eating disorder patients to use, more mature versions of the app should be evaluated by the intended user group. Another potential weakness was that the heuristic evaluation was translated from English to Swedish but has not been validated in the translated language. Owing to the early stage of development, most mERA items could not be objectively evaluated, but instead an opinion was sought from participants. This is not the intended use of the mERA checklist, which could lead to reduced reproducibility.

Future studies should aim to evaluate usability in eating disorder patients, investigate if VR is effective in changing behavior, and implement the app in an intervention. To ensure scalability to other clinics, especially those less experienced with handling electronic devices as a tool to help treat eating disorders, a clear protocol should be established, similar to that currently existing for handling a medical device (Mandometer) in eating disorder clinics [15]. Due to the immature state of VR as a treatment method, more general studies should also be performed to evaluate how various elements such as modes of information transfer (ie, tactile, visual, and auditory) influence usability and compliance.

The app was found to be usable and eating disorder personnel were positive regarding its incorporation in treatment. The app would fit well with current treatment, requiring only minor alterations. The overall consensus was that the app should be introduced at the beginning of treatment, be administered in parallel with real-life training, technical support should be available, and initial app use should be restricted to the clinic. Along with the food type dimension, there were requests by eating disorder personnel to allow changes to the environment and social context (eg, crowded room vs eating alone).

## Appendix

Multimedia Appendix 1 Tasks performed in the app by participants.

## Abbreviations

IT: information technology
mERA: mHealth Evidence and Reporting Assessment
SUS: System Usability Scale
VR: virtual reality

## References

[1] American Psychiatric Association (2013). Diagnostic Statistical Manual of Mental Disorders, 5th Edition.

[2] Hoek HW, van Hoeken D (2003). Review of the prevalence and incidence of eating disorders. Int J Eat Disord.

[3] Udo T, Grilo CM (2018). Prevalence and correlates of DSM-5-defined eating disorders in a nationally representative sample of U.S. adults. Biol Psychiatry.

[4] Arcelus J, Mitchell AJ, Wales J, Nielsen S (2011). Mortality rates in patients with anorexia nervosa and other eating disorders. A meta-analysis of 36 studies. Arch Gen Psychiatry.

[5] Smink FRE, van Hoeken D, Hoek HW (2012). Epidemiology of eating disorders: incidence, prevalence and mortality rates. Curr Psychiatry Rep.

[6] Zipfel S, Löwe B, Reas DL, Deter HC, Herzog W (2000). Long-term prognosis in anorexia nervosa: lessons from a 21-year follow-up study. Lancet.

[7] Dobrescu SR, Dinkler L, Gillberg C, Råstam M, Gillberg C, Wentz E (2020). Anorexia nervosa: 30-year outcome. Br J Psychiatry.

[8] Westmoreland P, Krantz MJ, Mehler PS (2016). Medical complications of anorexia nervosa and bulimia. Am J Med.

[9] Steinhausen H (2002). The outcome of anorexia nervosa in the 20th century. Am J Psychiatry.

[10] Treasure J, Duarte TA, Schmidt U (2020). Eating disorders. Lancet.

[11] Södersten P, Bergh C, Leon M, Brodin U, Zandian M (2017). Cognitive behavior therapy for eating disorders versus normalization of eating behavior. Physiol Behav.

[12] Wallier J, Vibert S, Berthoz S, Huas C, Hubert T, Godart N (2009). Dropout from inpatient treatment for anorexia nervosa: critical review of the literature. Int J Eat Disord.

[13] Fichter MM, Quadflieg N, Crosby RD, Koch S (2017). Long-term outcome of anorexia nervosa: Results from a large clinical longitudinal study. Int J Eat Disord.

[14] Bergh C, Callmar M, Danemar S, Hölcke M, Isberg S, Leon M, Lindgren J, Lundqvist A, Niinimaa M, Olofsson B, Palmberg K, Pettersson A, Zandian M, Asberg K, Brodin U, Maletz L, Court J, Iafeta I, Björnström M, Glantz C, Kjäll L, Rönnskog P, Sjöberg J, Södersten P (2013). Effective treatment of eating disorders: results at multiple sites. Behav Neurosci.

[15] Esfandiari M, Papapanagiotou V, Diou C, Zandian M, Nolstam J, Södersten P, Bergh C (2018). Control of eating behavior using a novel feedback system. J Vis Exp.

[16] Murray SB, Strigo IA (2018). Anorexia nervosa, neuroimaging research, and the contextual salience of food cues: The food approach-avoidance conundrum. Int J Eat Disord.

[17] Clus D, Larsen ME, Lemey C, Berrouiguet S (2018). The use of virtual reality in patients with eating disorders: systematic review. J Med Internet Res.

[18] Ferrer-García M, Gutiérrez-Maldonado J (2012). The use of virtual reality in the study, assessment, and treatment of body image in eating disorders and nonclinical samples: a review of the literature. Body Image.

[19] Maples-Keller JL, Bunnell BE, Kim S, Rothbaum BO (2017). The use of virtual reality technology in the treatment of anxiety and other psychiatric disorders. Harv Rev Psychiatry.

[20] Gorini A, Riva G (2008). Virtual reality in anxiety disorders: the past and the future. Expert Rev Neurother.

[21] de Carvalho MR, Dias TR, Duchesne M, Nardi AE, Appolinario JC (2017). Virtual reality as a promising strategy in the assessment and treatment of bulimia nervosa and binge eating disorder: a systematic review. Behav Sci (Basel).

[22] Gorini A, Griez E, Petrova A, Riva G (2010). Assessment of the emotional responses produced by exposure to real food, virtual food and photographs of food in patients affected by eating disorders. Ann Gen Psychiatry.

[23] Ferrer-García M, Gutiérrez-Maldonado J (2012). The use of virtual reality in the treatment of eating disorders. Stud Health Technol Inform.

[24] Toro J, Cervera M, Feliu MH, Garriga N, Jou M, Martinez E, Toro E (2003). Cue exposure in the treatment of resistant bulimia nervosa. Int J Eat Disord.

[25] Garcia-Palacios A, Botella C, Hoffman H, Fabregat S (2007). Comparing acceptance and refusal rates of virtual reality exposure vs. in vivo exposure by patients with specific phobias. Cyberpsychol Behav.

[26] Hoffmann TC, Glasziou PP, Boutron I, Milne R, Perera R, Moher D, Altman DG, Barbour V, Macdonald H, Johnston M, Lamb SE, Dixon-Woods M, McCulloch P, Wyatt JC, Chan A, Michie S (2014). Better reporting of interventions: template for intervention description and replication (TIDieR) checklist and guide. BMJ.

[27] Agarwal S, LeFevre AE, Lee J, L'Engle K, Mehl G, Sinha C, Labrique A, WHO mHealth Technical Evidence Review Group (2016). Guidelines for reporting of health interventions using mobile phones: mobile health (mHealth) evidence reporting and assessment (mERA) checklist. BMJ.

[28] Maramba I, Chatterjee A, Newman C (2019). Methods of usability testing in the development of eHealth applications: A scoping review. Int J Med Inform.

[29] Zapata BC, Fernández-Alemán JL, Idri A, Toval A (2015). Empirical studies on usability of mHealth apps: a systematic literature review. J Med Syst.

[30] Brown W, Yen P, Rojas M, Schnall R (2013). Assessment of the Health IT Usability Evaluation Model (Health-ITUEM) for evaluating mobile health (mHealth) technology. J Biomed Inform.

[31] Huryk LA (2010). Factors influencing nurses' attitudes towards healthcare information technology. J Nurs Manag.

[32] Jordan PW, Thomas B, Weerdmeester BA, McClelland IL (1996). SUS - a quick and dirty usability scale. Usability Evaluation In Industry.

[33] Andre TS, Hartson HR, Williges RC (2003). Determining the effectiveness of the usability problem inspector: a theory-based model and tool for finding usability problems. Hum Factors.

[34] Weinschenk D, Barker DT (2000). Designing effective speech interfaces.

[35] Bangor A, Kortum PT, Miller JT (2008). An empirical evaluation of the System Usability Scale. Int J Human-Comput Interact.

[36] Moncher FJ, Prinz RJ (1991). Treatment fidelity in outcome studies. Clin Psych Rev.

[37] Quirk J, Fish DR, Smith SJ, Sander JW, Shorvon SD, Allen PJ (1995). Incidence of photosensitive epilepsy: a prospective national study. Electroencephalogr Clin Neurophysiol.

[38] Ferrer-García M, Gutiérrez-Maldonado J, Pla-Sanjuanelo J, Vilalta-Abella F, Riva G, Clerici M, Ribas-Sabaté J, Andreu-Gracia A, Fernandez-Aranda F, Forcano L, Riesco N, Sánchez I, Escandón-Nagel N, Gomez-Tricio O, Tena V, Dakanalis A (2017). A randomised controlled comparison of second-level treatment approaches for treatment-resistant adults with bulimia nervosa and binge eating disorder: assessing the benefits of virtual reality cue exposure therapy. Eur Eat Disord Rev.

