# Correction: Virtual Reality App for Treating Eating Behavior in Eating Disorders: Development and Usability Study

**DOI:** 10.2196/29686

**Published:** 2021-04-20

**Authors:** Billy Sundström Langlet, Dorothy Odegi, Modjtaba Zandian, Jenny Nolstam, Per Södersten, Cecilia Bergh

**Affiliations:** 1 Division of Clinical Geriatrics, Center for Alzheimer Research Department of Neurobiology, Care Sciences and Society Karolinska Institutet Stockholm Sweden; 2 Mandometer Clinic Stockholm Sweden

In “Virtual Reality App for Treating Eating Behavior in Eating Disorders: Development and Usability Study” (JMIR Serious Games 2021;9(2):e24998) the authors noted two errors.

Due to a system error, the name of one author, Dorothy Odegi, was replaced with the name of another author on the paper, Jenny Nolstam. In the originally published paper, the order of authors was listed as follows:

Billy Sundström Langlet; Jenny Nolstam; Modjtaba Zandian; Jenny Nolstam; Per Södersten; Cecilia Bergh

This has been corrected to:

Billy Sundström Langlet; Dorothy Odegi; Modjtaba Zandian; Jenny Nolstam; Per Södersten; Cecilia Bergh

In the originally published paper, the ORCID of author Jenny Nolstam was incorrectly published as follows:

0000-0001-6120-9412

This has been corrected to:

0000-0002-9928-2778

The correction will appear in the online version of the paper on the JMIR Publications website on April 20, 2021, together with the publication of this correction notice. Because this was made after submission to PubMed, PubMed Central, and other full-text repositories, the corrected article has also been resubmitted to those repositories.

