# Supplementary material for: Virtual Reality App for Treating Eating Behavior in Eating Disorders: Development and Usability Study
Source: JMIR Serious Games. 2021 Apr 13;9(2):e24998. doi: 10.2196/24998 (PMC8057519; doi:10.2196/24998)

Tasks of the Virtual Reality app

The following figures show the tasks in the order they were performed by participants in the study. Red arrows in figures have been added for emphasis and were not displayed in the Virtual Reality app. The actions described after the task number was what was evaluated in the cognitive walkthrough. Interactable objects in the tutorial and while serving food were outlined in teal in the Virtual Reality app.

Tutorial

1. START - Look at the ‘START TUTORIAL’ button to start the App.

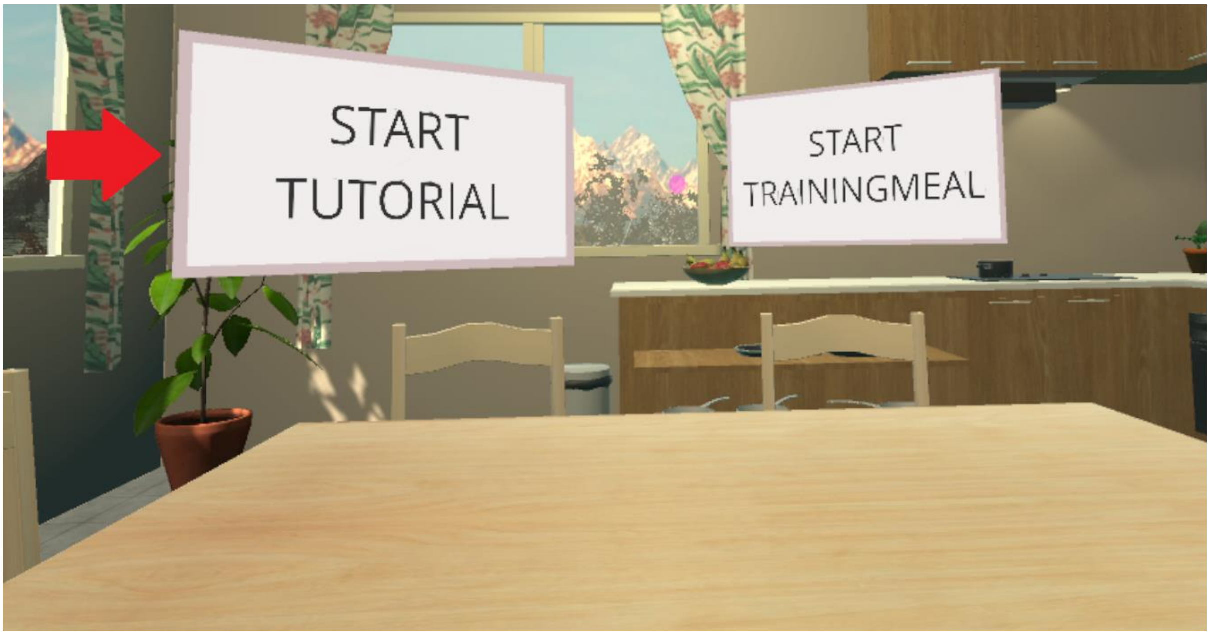

2. CONTROLLER INSTRUCTIONS - Read instructions on the tablet and click the ‘Next’ button using the controller.

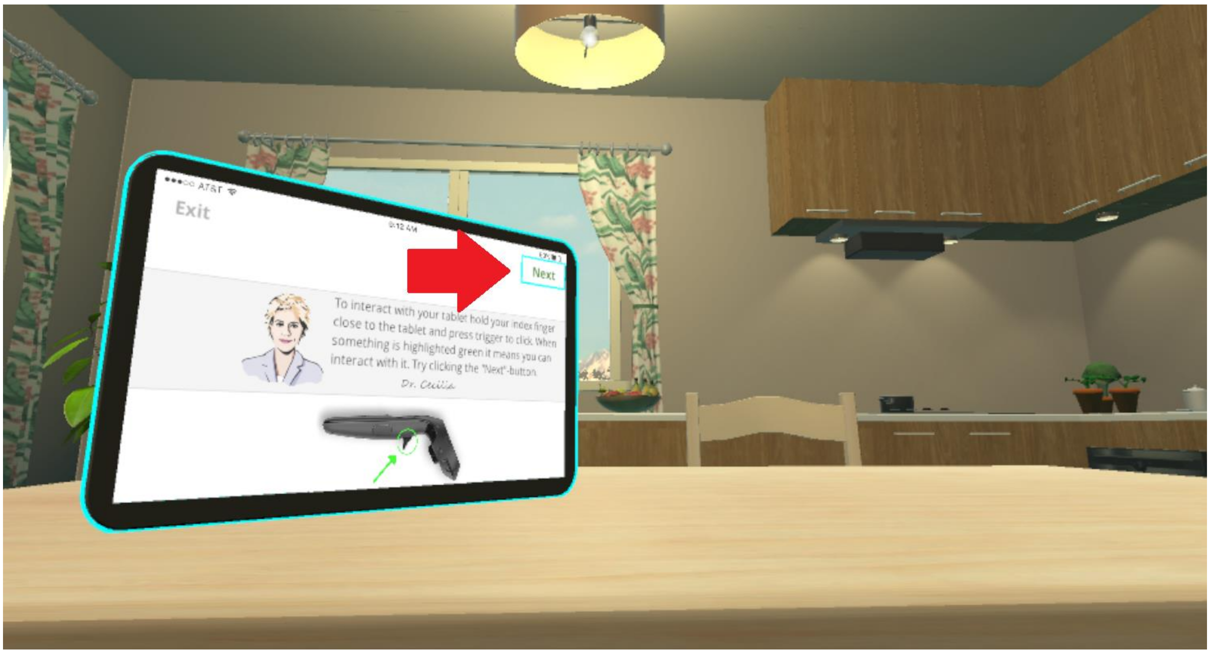

3. SERVING MEATBALLS - Read instructions then serve meatballs using the spoon. Click ‘Next’ when finished.

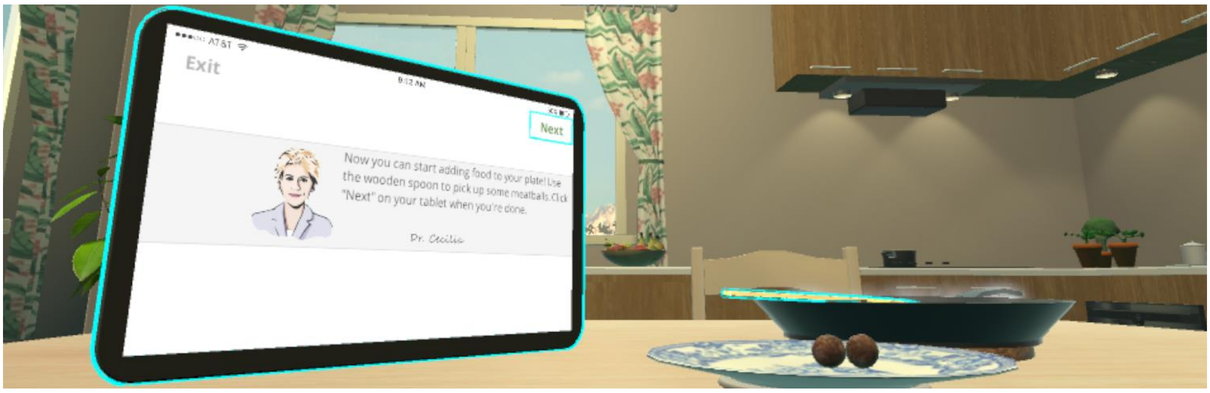

4. SERVING POTATOES - Read instructions then serve potatoes using the fork. Click ‘Next’ when finished.

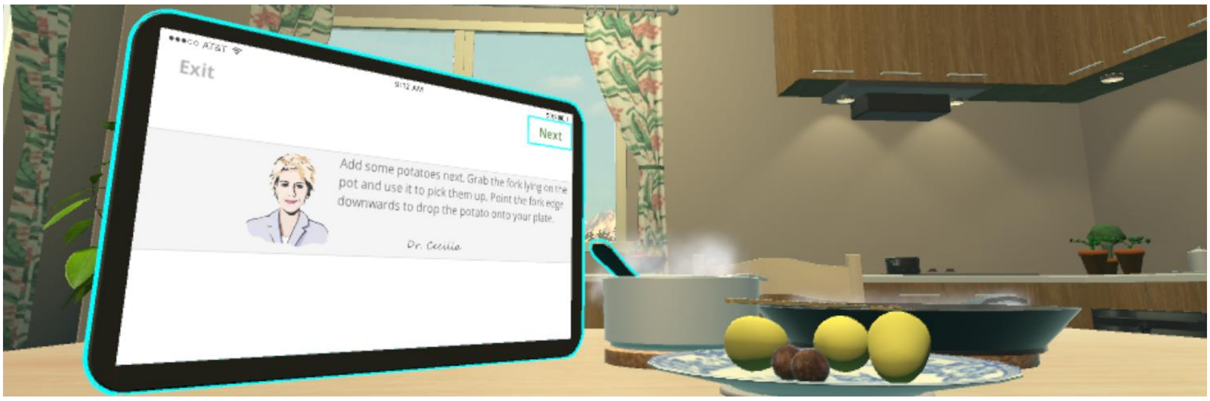

5. SERVING SOUP - Read instructions then serve gravy using the soup boat. Click ‘Next’ when finished.

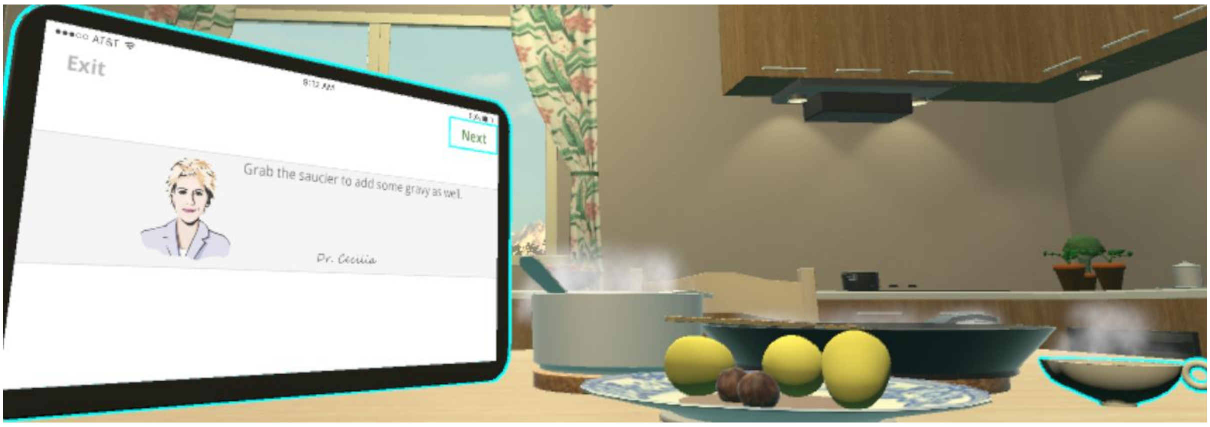

6. SERVING LINGONBERRY - Read instructions then serve lingonberry using the spoon. Click 'Next' when finished.

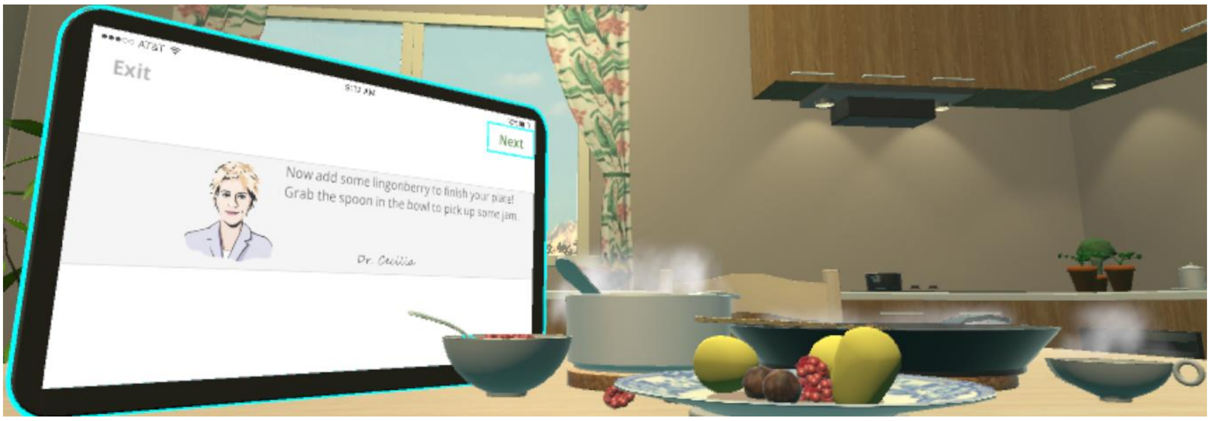

7. SERVING WATER - Read instructions then pour water from the jug into the glass. Click 'Next' when finished.

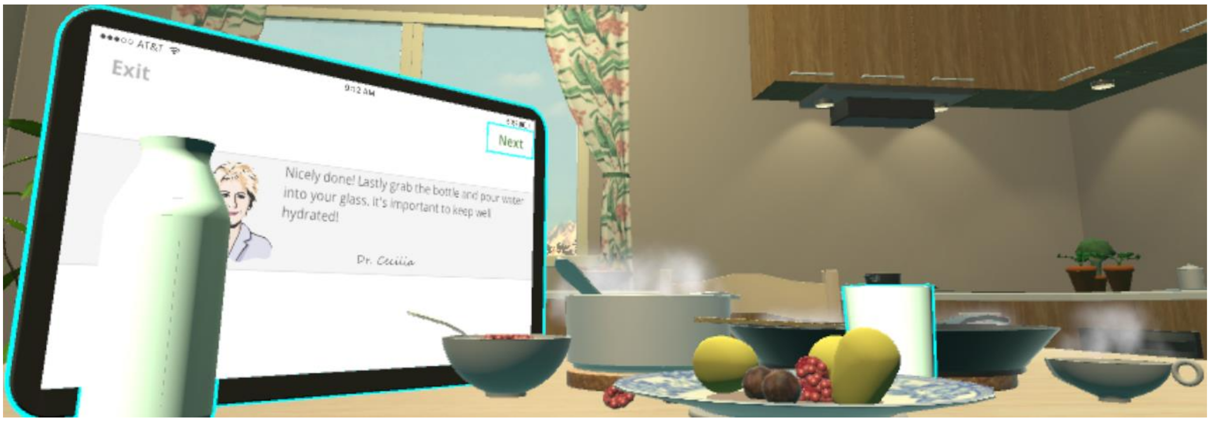

8. EAT FOOD - Read the instructions then manipulate food (cut potatoes into pieces), pick up and eat food. Click 'Next' when finished.

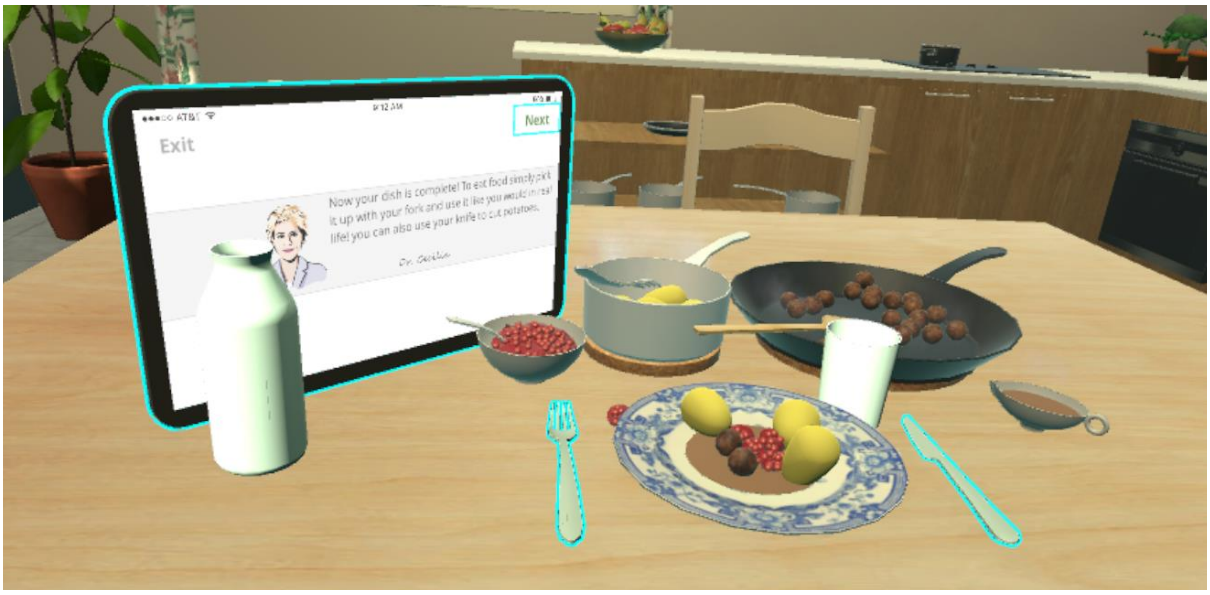

Eat training

1. START - Look at the 'START TRAININGMEAL' button to start the App.

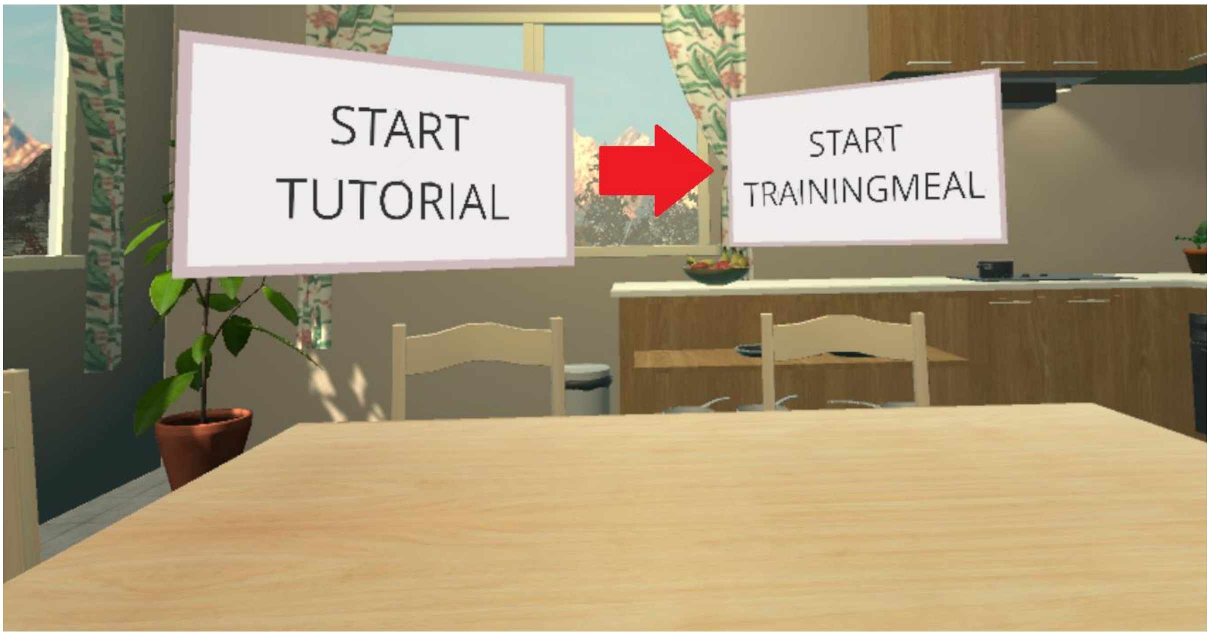

2. SERVING FOOD - Read instructions then serve food until the tablet shows 100%. Click 'Next' when finished

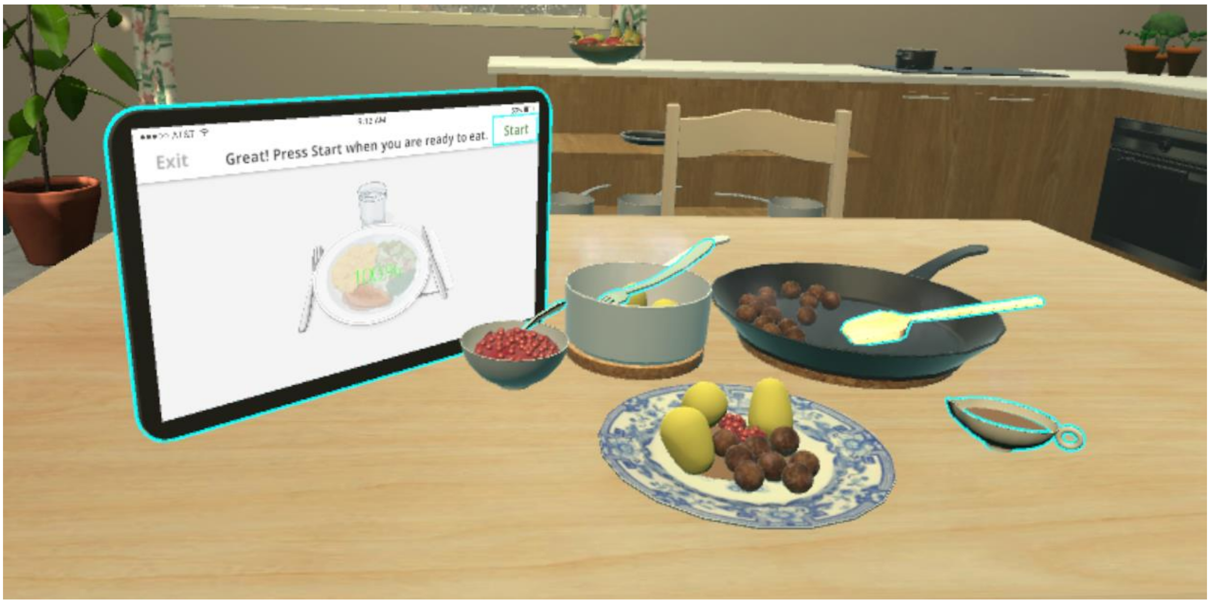

3. EAT TRAINING - Follow the training curve by either slowing down or increasing speed of eating.

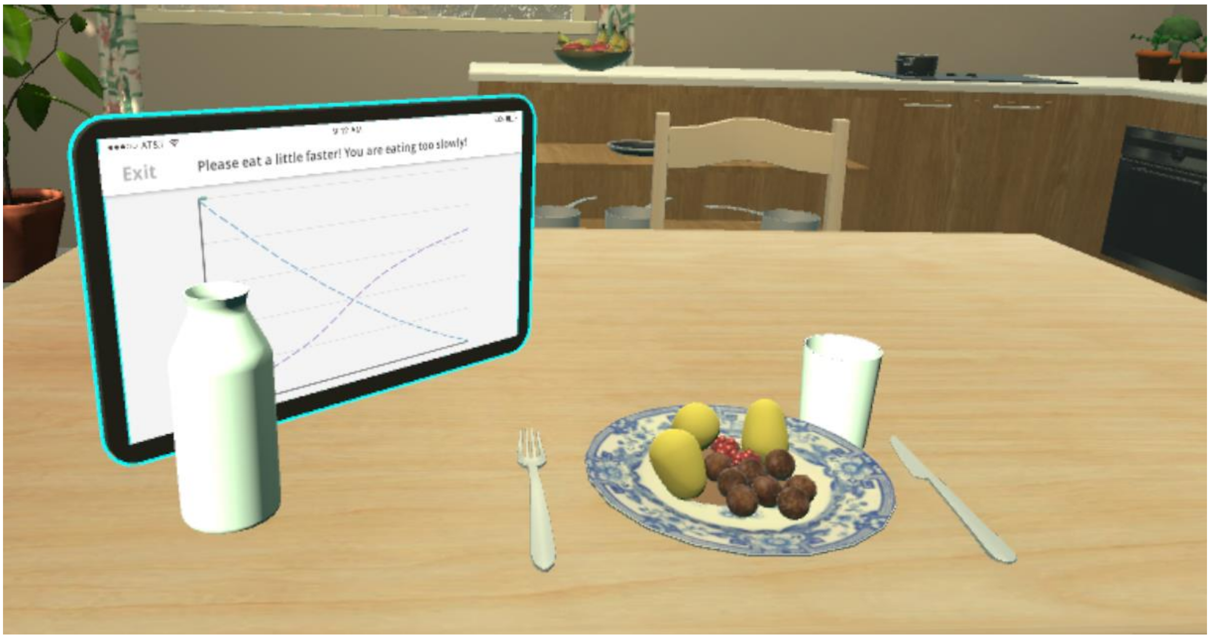

4. EXITING – When finished eating press the 'Restart' button to get back to the main menu.

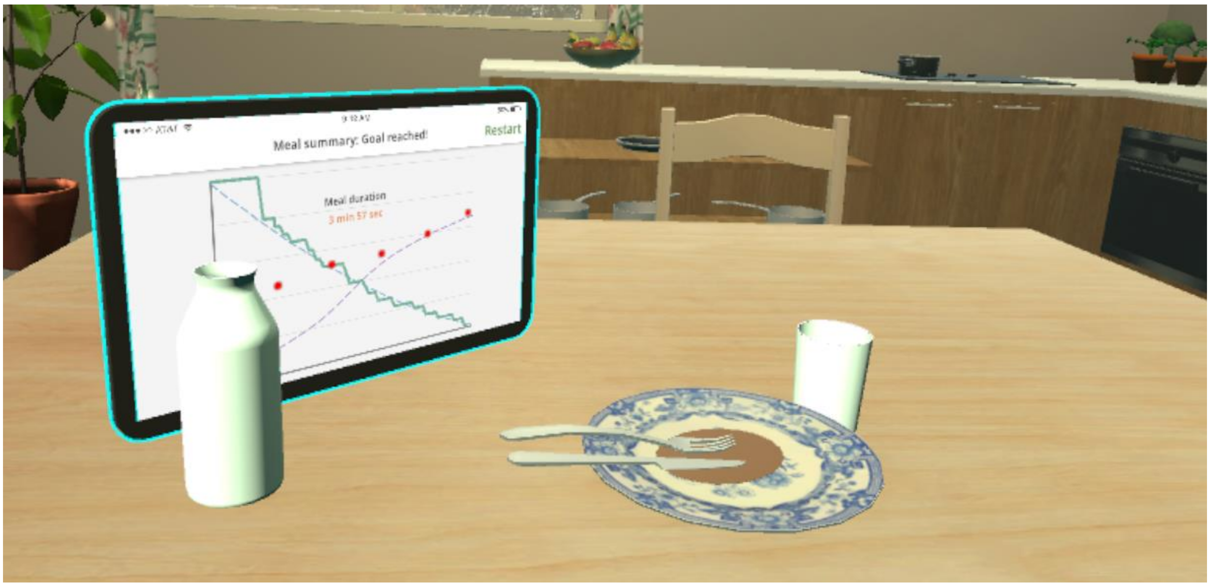

Supplement: Multimedia Appendix 1 [file games_v9i2e24998_app1.pdf]
